# Multi-Capillary Column-Ion Mobility Spectrometry of Volatile Metabolites Emitted by *Saccharomyces Cerevisiae*

**DOI:** 10.3390/metabo4030751

**Published:** 2014-09-05

**Authors:** Christoph Halbfeld, Birgitta E. Ebert, Lars M. Blank

**Affiliations:** iAMB—Institute of Applied Microbiology, ABBt—Aachen Biology and Biotechnology, RWTH Aachen University, Worringer Weg, Aachen 52074, Germany; E-Mails: christoph.halbfeld@rwth-aachen.de (C.H.); lars.blank@rwth-aachen.de (L.M.B.)

**Keywords:** volatile metabolites, VOC, *Saccharomyces cerevisiae*, yeast, ion mobility spectrometry, MCC-IMS, real-time fermentation monitoring, metabolism

## Abstract

Volatile organic compounds (VOCs) produced during microbial fermentations determine the flavor of fermented food and are of interest for the production of fragrances or food additives. However, the microbial synthesis of these compounds from simple carbon sources has not been well investigated so far. Here, we analyzed the headspace over glucose minimal salt medium cultures of *Saccharomyces cerevisiae* using multi-capillary column-ion mobility spectrometry (MCC-IMS). The high sensitivity and fast data acquisition of the MCC-IMS enabled online analysis of the fermentation off-gas and 19 specific signals were determined. To four of these volatile compounds, we could assign the metabolites ethanol, 2-pentanone, isobutyric acid, and 2,3-hexanedione by MCC-IMS measurements of pure standards and cross validation with thermal desorption–gas chromatography-mass spectrometry measurements. Despite the huge biochemical knowledge of the biochemistry of the model organism *S. cerevisiae*, only the biosynthetic pathways for ethanol and isobutyric acid are fully understood, demonstrating the considerable lack of research of volatile metabolites. As monitoring of VOCs produced during microbial fermentations can give valuable insight into the metabolic state of the organism, fast and non-invasive MCC-IMS analyses provide valuable data for process control.

## 1. Introduction

Yeasts are key model organisms in eukaryotic research and play a significant role in many biotechnological processes. The use of yeast for biotechnological processes dates back to 7000–5000 BC, where it has been used for wine fermentation and in food processing. [1,2,3,4]. Today, yeast strains are used in the field of industrial, food, and pharmaceutical industry for the synthesis of broad range of products ranging from bakery products to bioethanol and pharmaceuticals [5,6,7,8]. With 4 million tons of yeast biomass produced worldwide in 2009 and an estimated yearly increase of 7% [9], the yeast biotechnological market is economically significant and growing. The so far most often used yeast cell factory is the baker’s yeast *Saccharomyces cerevisiae*. This yeast is not only the most widely used yeast in biotechnology, but is additionally the model system for eukaryotic organisms. Accordingly, much research has been conducted with *S. cerevisiae* and many of the today state-of-the-art analytical techniques and molecular biology methods have been first developed for and with this yeast. The simple and rapid cultivation, genetic accessibility, and industrial importance were and still are drivers to maintain its lead in the yeast community in many disciplines of science.

The haploid *S. cerevisiae* genome consists of about 12,500 kb and was completely sequenced as early as 1996 (first complete genome sequence of a eukaryote) [10]. Physiological and functional genomics studies have characterized 5097 of the 6607 open reading frames (ORFs) [11]. The genomic and metabolic information is gathered in different databases (SGD [12], MIPS [13], YPD [14]) and in genome-scale metabolic network reconstructions. The first genome-scale metabolic model was published in 2003 [15], which consisted of 1175 metabolic reactions and 584 metabolites. Since then, several revised metabolic yeast models were reported (e.g., [16,17,18,19]), which were for example extended by the intracellular location of the respective metabolic pathways, *i.e.*, by compartmentation, and by the ever-increasing knowledge generated by the yeast community. A comprehensive consensus model was assembled in 2008 [20], which is continually updated. Its current version [21] comprises 2220 metabolites participating in 3490 reactions, which are annotated with 910 yeast genes encoding the catalyzing enzymes, about 18% of the verified open reading frames included in the *Saccharomyces* Genome Database [12]. Also, a lot of effort is put into the integration of different omics data in order to get a quantitative understanding of the metabolism and its regulation [22].

Surprisingly, the volatile metabolites produced by yeast are mainly neglected in yeast research with the most prominent exceptions of acetate and ethanol. This can be attributed to the limited knowledge of the biochemistry and genetics involved in the formation of volatile metabolites that hinder the general incorporation in systems biology studies. By far, most reports of volatile metabolites from yeast-derived products originate from researchers interested in high quality wine making [23,24,25,26,27], as the scent of wine impacts its organoleptic properties [28]. The main classes of volatile metabolites observed are alcohols, aldehydes, and esters (Table 1); while depending on grape and yeast used, many others can be found. Notably, most studies reported were carried out on media that have wine-like compositions. Thus, the media are most often complex, with alternative carbon and nitrogen sources present. The observed volatile metabolites can therefore originate from glucose catabolism or are products from biotransformation, *i.e.*, do not originate from sugar (the carbon and energy source), but rather from grape metabolites that were only modified by yeast enzymes.

Recently, the known microbial volatile organic compounds (mVOCs) have been gathered in a database, mVOC [29], containing 846 metabolites from 349 bacterial and 69 fungal species (as of December 2013). Compared to other VOC specific databases such as Pherobase [30], SuperScent [31] or FlavorNet [32] or the recently published compilation of VOCs from the human body [33], which list up to 8000 compounds, the volatile metabolite space of microbes is rather little explored. This becomes all the more apparent, when checking the representation of VOCs in metabolic databases or genome-scale metabolic reconstructions. Of the 93 compounds listed in Table 1, only 19 are contained in the current yeast genome-scale metabolic model (Version 7.11) [34] or the yeast genome database SGD [12].

**Table 1:** Volatile organic compounds emitted from *S. cerevisiae* fermentations. It is indicated which of the compounds are included in the latest yeast genome scale metabolic reconstruction (Yeast 7.11) and in the *Saccharomyces* genome database SGD.

Compound	Compound class	PubChem ID	Included in		Reference ^#^
			Yeast 7.11	SGD	
(2-phenylcyclopropyl) methanol	alcohols	317540	no	no	[35]
1,2-benzenedicarboxylic acid	acids	1017	no	no	[29,36]
1,3-butanediol	alcohols	6440	no	no	[35]
1-butanol	alcohols	263	no	no	[35]
1-heptanol	alcohols	8129	no	no	[35]
1-hexanol	alcohols	8103	no	no	[23,24,25,37]
1-propanol	alcohols	1031	no	no	[23,24,25,37,38]
2,3-butanediol	alcohols	262	yes	yes	[23,24]
2,5-dimethylpyrazine *	pyrazines	31252	no	no	[29,36]
2-ethyl-1-hexanol	alcohols	7720	no	no	[29,36,37]
2-furfuraldehyde	aldehydes	7362	no	no	[25]
2-hexanol	alcohols	12297	no	no	[35]
2-methyl-2-butanol *	alcohols	6405	no	no	[24,25,37]
2-methylbutanal *	aldehydes	7284	yes	no	[29,36]
2-methylbutanoic acid	acids	8314	no	no	[29,36]
2-methylbutanol	alcohols	8723	yes	yes	[24,25,37]
2-pentanone	ketones	7895	no	no	[29,36]
2-phenylethanol *	benzenoids	6054	yes	yes	[23,24,25,26,29,36]
2-phenylethyl acetate	esters	7654	no	no	[25,26]
2-propane	alkanes	6334	no	no	[37]
2-propanol	alcohols	3776	no	no	[29,36,37,38]
2-xylene	benzenoids	7237	no	no	[29,36]
3-methylbutanal *	aldehydes	11552	yes	yes	[37]
3-methylbutanoic acid *	acids	10430	no	no	[24,25,29,36]
3-methylheptyl acetate	esters	537686	no	no	[37]
5-methyl-2-furfuraldehyde	aldehydes	12097	no	no	[25]
acetaldehyde	aldehydes	177	yes	yes	[24,25,37,38]
acetaldehyde diethylacetal	ethers	7765	no	no	[25]
acetic acid	acids	176	yes	yes	[23,29,36]
acetic acid 2-propenyl ester	esters	11584	no	no	[37]
acetic acid ethenyl ester	esters	7904	no	no	[29,36]
acetoin	ketones	179	yes	yes	[25]
acetone	ketones	180	no	no	[29,36]
acetophenone	ketones	7410	no	no	[25]
benzaldehyde	aldehydes	240	no	no	[25]
benzyl acetate	alcohols	8455	no	no	[25]
benzyl alcohol *	alcohols	244	no	yes	[25]
butanal	aldehydes	261	no	no	[37]
butanone *	ketones	6569	no	no	[29,36,37]
butyric acid	carboxylic acids	264	no	no	[24,26]
cis-3-hexen-1-ol	alcohols	5281167	no	no	[24,25]
decanoic acid	carboxylic acids	2969	no	no	[23,24,25,26]
diacetyl *	ketones	650	no	yes	[25,37]
diethyl succinate	esters	31249	no	no	[23,24,25]
dimethyl disulfide *	sulfides	12232	no	no	[29,36]
dodecanoic acid *	carboxylic acids	3893	no	no	[24,26]
ethanol	alcohols	702	yes	yes	[29,36]
ethyl 2-methylbutyrate	esters	24020	no	no	[25]
ethyl acetate	esters	8857	yes	no	[24,25,29,36,37,38]
ethyl benzoate	esters	7165	no	no	[25]
ethyl butyrate	esters	7762	no	no	[23,25,26]
ethyl caproate	esters	31265	no	no	[23,24,25,26,37]
ethyl caprylate	esters	7799	no	no	[23,24,25,26,37]
ethyl decanoate	esters	8048	no	no	[23,24,25,26]
ethyl furoate	esters	11980	no	no	[25]
ethyl heptanoate	esters	7797	no	no	[25]
ethyl isobutyrate	esters	7342	no	no	[25,37]
ethyl isovalerate	esters	7945	no	no	[25,37]
ethyl lactate	esters	7344	no	no	[24,25]
ethyl pehnylacetate	esters	7590	no	no	[25]
ethyl propanoate	esters	7749	no	no	[25,37]
ethyl pyruvate	esters	12041	no	no	[24]
ethyl valerate	esters	10882	no	no	[25]
ethyl-2-hydroxy propionate	esters	545098	no	no	[23]
furfuryl alcohol *	alcohols	7360	no	no	[25]
guaiacol *	alcohols	460	no	yes	[25]
heptanal *	aldehydes	8130	no	no	[37]
heptanoic acid	carboxylic acids	8094	no	no	[25]
hexanal	aldehydes	6184	no	no	[25]
hexanoic acid *	carboxylic acids	8892	no	no	[23,24,25,26]
hexyl acetate	esters	8908	no	no	[24,25,26]
isoamyl acetate	esters	31276	yes	no	[37]
isoamyl alcohol	alcohols	31260	yes	yes	[23,24,25,26,37]
isobutanal *	aldehydes	6561	no	yes	[37]
isobutanol	alcohols	6560	yes	yes	[23,24,25,29,36,37,38]
isobutyl acetate	esters	8038	yes	no	[25,37]
limonene	terpenes	22311	no	no	[29,36]
linalyl propionate	esters	61098	no	no	[23]
methanol *	alcohols	887	no	no	[24,25]
methyl acetate	silanes	76214	no	no	[25]
methylpropanoic acid *	acids	6590	no	yes	[24,29,36]
monoethyl succinate	esters	70610	no	no	[23]
n-butyl acetate	esters	31272	no	no	[25,37]
nonanal	aldehydes	31289	no	no	[37]
nonanoic acid	carboxylic acids	8158	no	no	[25]
n-propyl acetate	esters	7997	no	no	[25,37]
octanoic acid *	carboxylic acids	379	no	no	[23,24,25,26]
pentanal	aldehydes	8063	no	no	[37]
propionic acid	carboxylic acids	1032	no	no	[24]
pyrazine	pyrazines	9261	no	no	[29,36]
undecane	alkanes	14257	no	no	[29,36]
α-terpineol	terpenes	17100	no	no	[25]
β-phenylethyl formate	esters	7711	no	no	[37]

* These compounds were also detected in yeast extract [39]. In reference [36], yeast was cultivated in malt extract and tryptone soya, in reference [37] yeast extract, plus bactopeptone and glucose was used. All other references reported volatiles from wine fermentations.

**Figure 1:**
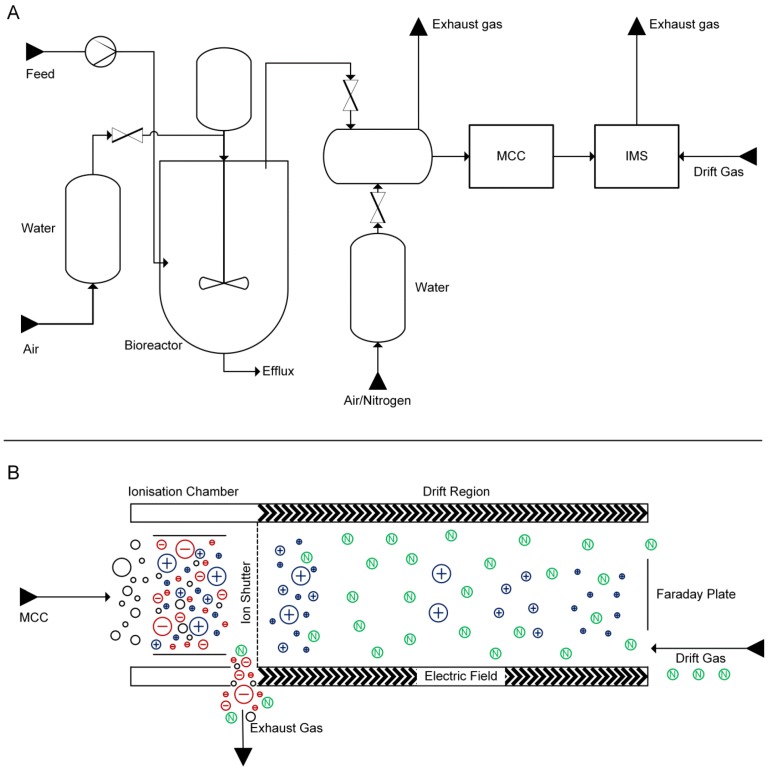
(**A**) Experimental set-up for the on-line MCC-IMS measurements of fermenter off-gas. (**B**) Working principle of the ion mobility spectrometer; adapted from [33].

One of the key bottlenecks in VOC research, and one of the reasons why these have not been studied broadly so far, is that sample preparation of gaseous chemicals requires additional care and that the analysis of volatiles is challenging. Most often, VOCs are extracted and enriched using head-space/solid phase microextraction (HS/SPME) methods and analyzed with gas chromatography coupled to advanced mass spectrometers [40,41]. An alternative technique for the analysis of volatile metabolites is ion mobility spectrometry (IMS). IMS has originally been used to detect explosives, chemical warfare agents or illegal drugs, but finds more and more broader applications, for example in medicine as diagnostic tool (breath analyses), for food quality control or monitoring and process control in the chemical and biotechnological industry. The increasing popularity can be attributed to its high sensitivity (detection limits down to ng L^−1^/pg L^−1^ or ppb_v_/ppt_v_ [42,43]), combined with relatively low investment and operating costs and high-speed data acquisition; a reading of a single spectrum takes only 20–50 ms [44]. The IMS separates analytes according to their gas-phase ion mobility. The sample is first ionized, e.g., directly by UV light or via charge transfer from ionized reactant ions produced by a radioactive ionization source (e.g., ^63^Ni). In the drift tube, the ionized molecules are accelerated by an electric field towards a Faraday plate, where the impact of the single ions is detected. While the ions are pulled along the drift tube they are separated by collision with the drift gas flowing into the opposite direction (Figure 1B). The ion mobility is compound specific and depends on the ion’s mass, charge, and shape. Hence, IMS allows the separation of molecule isomers.

The IMS is especially effective, when coupled to a multi capillary column (MCC) as a pre-separation unit. In this way, the volatile metabolites are separated in two dimensions, firstly according to the elution from the MCC and secondly through the specific drift velocity in the IMS. This hyphenated technique significantly increases the resolution of metabolites [40,45]. MCCs consists of approx. 1000 parallel capillaries that can withstand high gas fluxes and are insensitive to humidity levels up to 100% [46], thus allowing direct measurements of the fermentation off-gas without sample preparation. The total analysis time of one sample in an MCC-IMS is less than 500 s [44]. These characteristics together with the high sensitivity perfectly suit the MCC-IMS for on-line measurements of dilute volatile metabolites in the headspace of microbial fermentations. 

Mass spectrometers have a higher potential for identification of metabolites, but come with the disadvantage of longer time intervals for analysis. Furthermore, GC-MS instruments require special gases such as helium and high vacuum, hence come with relatively high operating costs and technical expenditure. In contrast, MCC-IMS can be operated with nitrogen or air (not necessarily synthetic air) and at ambient temperature and pressure. 

The potential of MCC-IMS analyses for fermentation monitoring has already been shown for measurements of mVOCs produced during batch cultivation of *Escherichia coli* and *Pseudomonas aeruginosa* in shake flasks [45,47]. Also, MCC-IMS measurements of yeast fermentation have been reported, in which on-line measurements of the off-gas of yeast fermentations were performed. While Kotiaho [48] focused on the quantification of one single metabolite, ethanol, Kohlemainen *et al.*, measured patterns of off-gas metabolites without any analyte identification [49]. The potential of IMS analyses for quality control during beer fermentation was shown by Vautz *et al.* [50] by monitoring the ripening indicators diacetyl and 2,3-pentanedione. In this contribution we aimed for online MCC-IMS measurements for the monitoring and, combined with GC-MS analyses, for the identification of mVOCs produced by *S. cerevisiae.* Different growth conditions were tested and special emphasis was put on the dynamics of the VOC profiles during transient metabolic conditions, *i.e.*, the shift from respiratory to fermentative metabolism. 

## 2. Results and Discussion

### 2.1. Experimental Setup

To enable off-gas measurements, the MCC-IMS had to be connected to the bioreactor in a controlled and robust manner. To achieve this, the off-gas of the bioreactor was connected to a mixing chamber where it was diluted with filtered, compressed air. To ensure proper mixing, the gas inlets were positioned at the bottom, while the gas outlet to the MCC-IMS was positioned in the upper section of the chamber. A second outlet leading through a filter into the environment was positioned at the top.

Between measurements, the MCC-IMS and sampling line were flushed with nitrogen at a flow rate of 100 mL min^−1^ (Figure 1A). To avoid microbial contaminations and the introduction of volatile impurities from the compressed air, the gas used for aeration of the bioreactor was passed through a 0.2 µm filter and a water bath. The overall setup allowed controlled sampling without disturbances from the environment as no impurities were observed in the MCC-IMS during abiotic operation of the bioreactor.

### 2.2. Growth-Dependent Production of Volatile Metabolites

#### 2.2.1. MCC-IMS Monitoring of Batch Cultures

**Figure 2:**
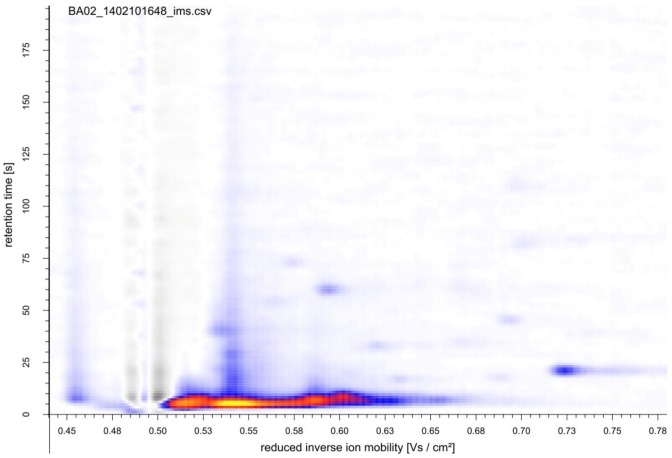
MCC-IMS topographic plot of sterile Verduyn medium. The reaction ion peak (1/K_0_ = 0.5 Vs cm^−2^) was compensated by the VisualNow software.

So far, most studies of yeast VOCs focused on the determination of volatiles produced during wine fermentation and their impact on wine aroma. These studies did not discriminate whether the VOCs originated from yeast fermentation or were the product of biotransformations of the grape constituents [23,24,25,26]. Here, to explore the mVOCs *de novo* synthesized by baker’s yeast, minimal salt medium with glucose as the sole carbon source was used. For a first evaluation of the MCC-IMS set-up for real-time fermentation monitoring, *S. cerevisiae* was grown in batch culture. Before inoculation of the fermenter, signals occurring from the sterile medium were measured under process conditions (stirrer speed, temperature and pH control, aeration). During a period of 12 h, the MCC-IMS measurements (Figure 2) showed four major signals of invariant intensity (data not shown).

**Figure 3:**
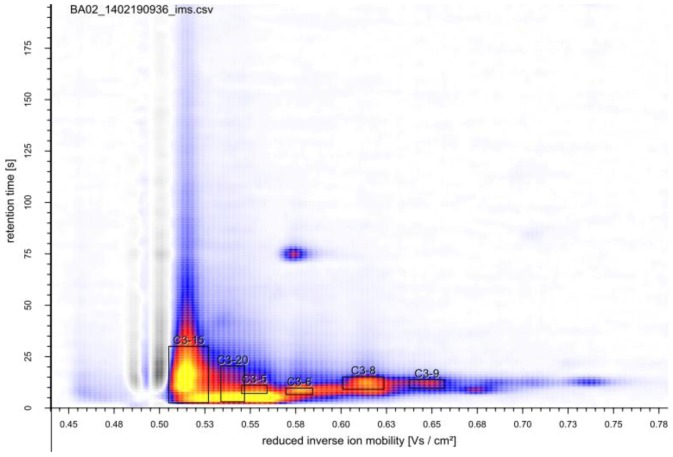
MCC-IMS topographic plot of *S. cerevisiae* in early stationary phase during batch fermentation. Boxes indicate analytes with the most significant changes during the growth (perturbation) experiments.

**Figure 4:**
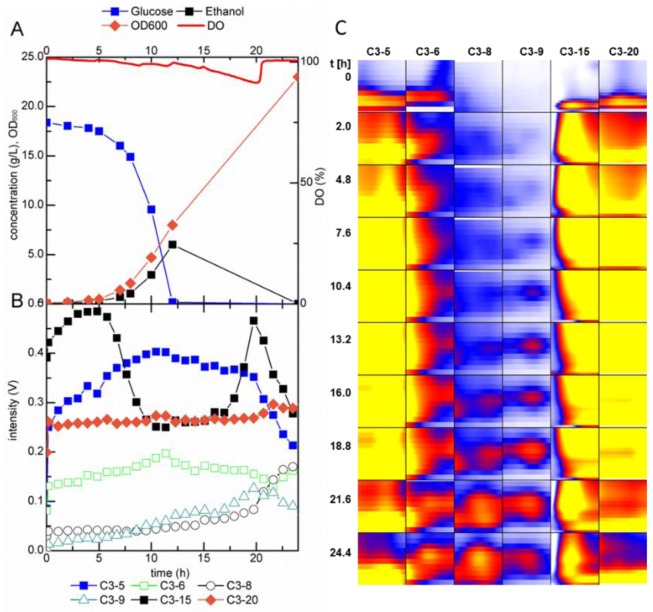
(**A**) Fermentation profile of *S. cerevisiae* during batch growth in glucose minimal medium, (**B**) trends in intensity, (**C**) heat map of selected peaks detected by MCC-IMS analysis of the fermentation off-gas. Areas in the heat map show the detected analyte peak and the surrounding area; DO = dissolved oxygen.

After inoculation to a starting OD_600_ of 0.1, MCC-IMS analyses were performed throughout the growth experiment in one hour intervals. In addition to the analytes detected in the sterile medium, 19 peaks emerged during the batch growth experiment, at retention times between 1 and 190 s (Figure 3). The time course of the six most distinctive peaks is shown in Figure 4B,C. To correlate the MCC-IMS patterns of the volatile metabolites to the yeast physiology, in parallel to the MCC-IMS measurements the optical cell density, carbon source consumption, and byproduct formation (ethanol, glycerol) were quantified (Figure 4A).

In the single fermentation experiment, the profile of signal C3-5 correlated with the growth rate since its signal increased after the lag phase and reached its maximum during the exponential growth phase. In the stationary phase, the peak intensity of C3-5 decreased again. Peak C3-9 was detected at the same time as ethanol, measured in the fermentation broth, and its signal diminished simultaneously with ethanol consumption. The signal of C3-8 showed an increasing trend in the late exponential to early stationary phase, when ethanol was nearly consumed. Signal C3-15 showed a rather peculiar profile. The signal intensity first increased, but rapidly decreased after 5 h and maintained a constant level for about 11 h. After this period, the intensity of C3-15 abruptly increased and reached its prior maximal value followed by a steady decrease at the end of the batch. This behavior might be explained by incomplete ionization of the analyte molecules. The signal of the two signals decreased when the peak of signal C3-5 increased. This signal might have a higher proton affinity and therefore be preferably ionized to substance C3-15. This hypothesis is substantiated by the low reaction ion peak (RIP) in the MCC-IMS chromatograms for the period between 5 h–21 h (data not shown). The RIP consists of reaction ion molecules originating from the drift gas, here nitrogen. In the ionization chamber, water molecules react with positively charged nitrogen ions to a cluster of the type (H_2_O)_n_H^+^. These ions form the RIP and transfer the charge to molecules with a higher proton affinity. Hence, with increasing analyte concentration the RIP diminishes. For more detailed information about the charge transfer reactions, the reader is referred to [51]. Note that the data presented here originate from single experiments and are thus not based on statistics. The intention of this work was the development of a set-up for online MCC-IMS measurements of volatile metabolites in the off-gas of yeast fermentations, with which we will generate more comprehensive datasets in future experiments.

To elucidate the potential of MCC-IMS analyses for the differentiation of different *S. cerevisiae* strains or mutants we compared the MCC-IMS chromatograms of *S. cerevisiae* CEN.PK 117 YOL086c::*kan*MX4, deficient in the major alcohol dehydrogenase Adh1p, with its isogenic reference strain. Again, we want to stress that in this proof-of-principle work, only single experiments were performed to show the general applicability of MCC-IMS for online measurements of fermentation off-gas. 

**Figure 5:**
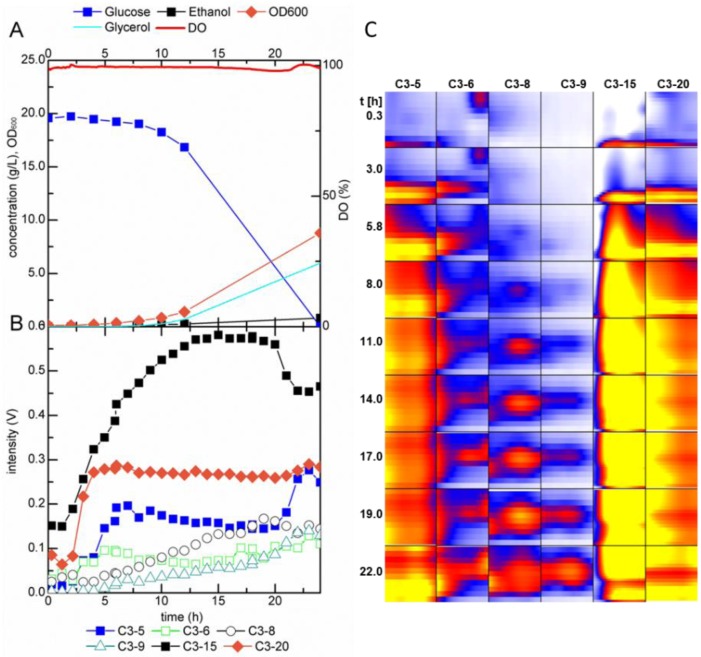
(**A**) Fermentation profile of *S. cerevisiae adh1*Δ during batch growth in glucose minimal medium and (**B**) trends in intensity and (**C**) heat map of selected peaks detected by MCC-IMS analysis of the fermentation off-gas. The areas in the heat map show the detected analyte peak and the surrounding area; DO = dissolved oxygen.

The *ADH1* deletion mutant had a reduced growth rate and biomass yield, about 56% and 42% of the reference values. The ethanol formation was clearly reduced (maximal accumulation of 0.8 g L^−1^ *vs*. 6 g L^−1^ for the reference strain) while glycerol production was increased (Figure 5A). These differences in the strain physiology were also reflected in the MCC-IMS pattern (Figure 5B,C). While no new peaks compared to the reference strain chromatograms were detected, the profiles of several peaks differed. Peak C3-15 increased much slower compared to the reference strain fermentation. The signal of C3-5 rose in the beginning, stagnated within the 5th–20th h after inoculation and increased afterwards. While the absolute intensity of peak C3-6 was lower in the measurements of the mutant strain compared to the reference strain, the time profile was similar for both cultivations., The profile of peak C3-20 was similar to that in the reference strain cultivation but showed a more shallow increase at the start of the fermentation while the intensity of C3-8 rose faster. Although replicate experiments are required for a statistically valid statement, we hypothesize that these distinct mVOC derived MCC-IMS signals allow differentiation of the two yeast strains. Similarly, species specific volatile footprints or markers have are already used to detect cancer via breath analysis of patients [52] or fungal contaminants in buildings [53].

These measurements gave first valuable information about the volatile metabolite patterns produced by yeast and their dynamics during growth on glucose minimal medium. However, because of the high signal of volatiles, most likely ethanol and acetaldehyde, in the off-gas and probable incomplete ionization of metabolites, data interpretation and thorough metabolite detection under these conditions was limited. 

#### 2.2.2. MCC-IMS Monitoring of Glucose-Limited Chemostat Fermentations

**Figure 6:**
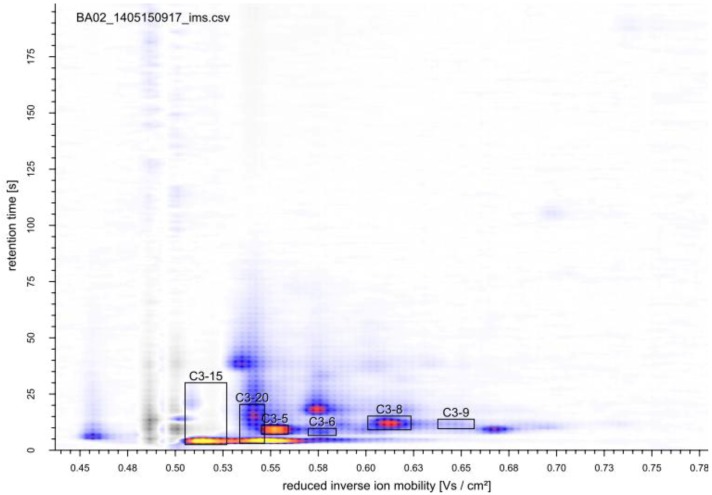
MCC-IMS topographic plot of the off-gas of a glucose-limited continuous cultivation of *S. cerevisiae*. Boxes indicate analytes with the most significant changes during the growth (perturbation) experiments. The reaction ion peak (1/K_0_ = 0.5 Vs cm^−2^) was compensated by the VisualNow software.

High glucose concentrations as in batch fermentations trigger glucose repression. One consequence is the induction of respiro-fermentative metabolism that is aerobic ethanol formation, known as Crabtree effect [54,55]. This switch of the metabolic mode is undesirable for example during production of yeast biomass or protein production, as ethanol formation reduces product yield and quality [56,57,58,59]. Such processes are therefore run as glucose-limited fermentations, in which this regulatory mechanism is repressed. While industrial processes are most often run as fed-batch fermentations, in academic research glucose-limited chemostats are favored as these continuous cultivations can be performed under defined, controlled, and constant conditions allowing very reproducible experiments. Moreover, this fermentation mode allows varying one single fermentation parameter, making it ideal for studying the impact of specific perturbations on growth physiology or metabolism. To elucidate possible changes in the volatile metabolites during the transition from respiratory to fermentative metabolism, we cultivated *S. cerevisiae* in a glucose-limited chemostat at a dilution rate of 0.13 h^−1^. Under these fully oxidative growth conditions, *S. cerevisiae* produced no ethanol. At metabolic steady state of the single experiment, the MCC-IMS chromatogram showed 13 peaks, which were not detected in the sterile medium, while two peaks observed in the sterile medium were not or with considerably less intensity detected in the chemostat culture (Figure 6). Because of the overloaded chromatogram during batch cultivation, it is difficult to state, which of these are specific for the glucose-limited respiratory growth conditions and which appear generally during growth of *S. cerevisiae*. The most distinct peaks were identical for both growth conditions. 

**Figure 7:**
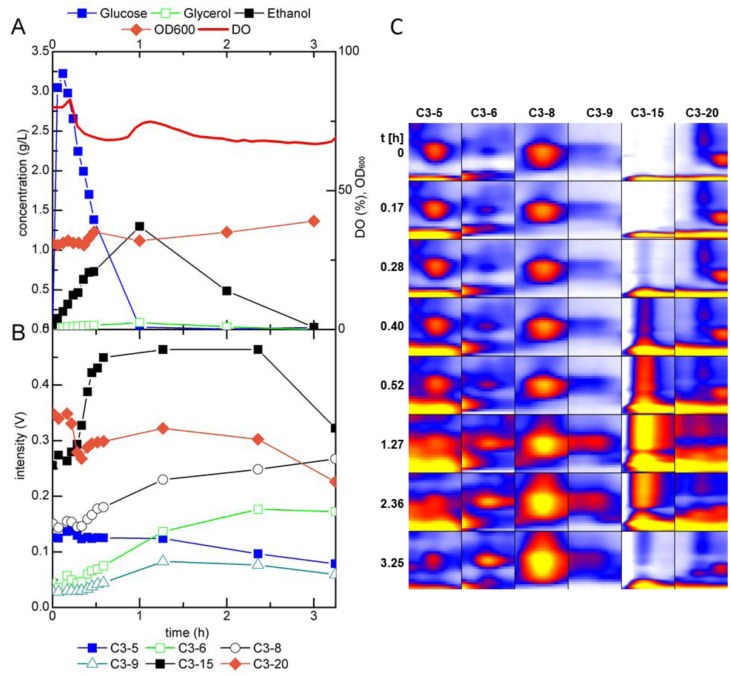
(**A**) Fermentation profile of *S. cerevisiae* during growth in a glucose-limited chemostat and (**B**) trends in intensity and (**C**) heat map of selected peaks detected by MCC-IMS measurements of the fermentation off-gas after perturbation of the metabolic steady state with a pulse of 22 mmol glucose; DO = dissolved oxygen.

To induce a shift from respiratory to fermentative metabolism, the steady state culture was perturbed once with a pulse of 22 mmol glucose, which was rapidly injected into the bioreactor. Immediately after the glucose pulse, ethanol accumulated in the fermentation broth (Figure 7A). Acetate (data not shown) and glycerol were detected as well and showed a similar profile as ethanol. The surplus glucose was consumed within 75 min, after which ethanol was catabolized and diminished about 3 h after the pulse. The optical density (OD_600_) increased from 31–39. The intensity of several analytes detected with the MCC-IMS increased after the pulse and decreased again after about 2 h (Figure 7B,C). The most prominent changes were observed for the analytes marked in Figure 6, these are the same peaks as in the batch cultivation of the wild type yeast and the ADH1 mutant. Peak C3-15 showed a strong correlation with the ethanol concentration determined in the fermentation broth. However, other peaks, like C3-6 and C3-8, did not resume the intensities prior to the perturbation, but maintained a higher level within the 7 h of MCC-IMS monitoring, hence, correlated with the increase in biomass concentration. C3-5 was the only peak whose intensity decreased upon glucose addition. About 3 h after the glucose pulse, its intensity increased again and regained its original value in the next 3 h. C3-20 showed a rapid decrease directly after the pulse, remained at a constant level and showed a decreasing trend after about 2 h and 30 min. 

**Figure 8:**
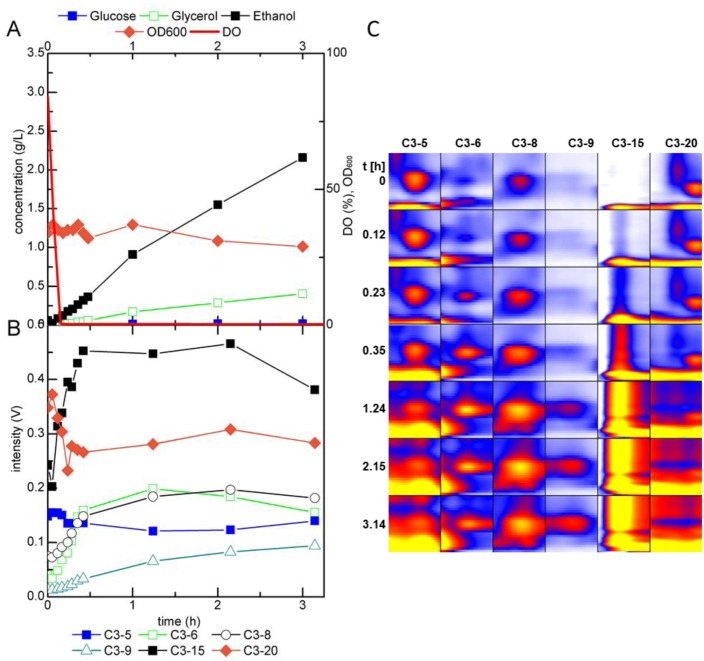
(**A**) Fermentation profile of *S. cerevisiae* during growth in a glucose-limited chemostat and (**B**) trends in intensity and (**C**) heat map of selected peaks detected by MCC-IMS measurements of the fermentation off-gas during transition to anaerobic conditions; DO = dissolved oxygen.

The physiological response of *S. cerevisiae* to limited oxygen availability is very similar to that of the Crabtree effect. In both cases, the fluxes through glycolysis are upregulated while the fluxes through the TCA are downregulated and as a consequence ethanol is produced [60,61]. To elucidate possible differences in the volatile metabolite patterns of yeast cultures responding to a glucose pulse and anaerobiosis, respectively, in a second perturbation experiment gassing was switched from air to nitrogen. In this single perturbation experiment, the dissolved oxygen (DO) concentration dropped to zero within 10 min. Simultaneously with this drop, ethanol and glycerol accumulated in the fermentation broth, while only little acetate, which accumulated only after 1 h of anaerobic growth (Figure 8A). The biomass concentration decreased slowly during anaerobiosis. Note, that the data of this perturbation experiment cannot be directly compared to the glucose pulse as the air supply was permanently replaced by the same flow rate of nitrogen. 

As in the glucose pulse perturbation experiment, peak C3-15 increased as soon as ethanol was produced (Figure 8B,C). The increase flattened after about 60 min and decreased after about 2 h Again incomplete ionization and possible incomplete evaluation of the peak area might be responsible for this trend. The intensities of peak C3-6 and C3-8 increased rapidly in the first 30 min after the shift to nitrogen gassing, considerably faster than in the glucose pulse experiment. C3-9 showed a slight increase while peak C3-5 stayed constant over the period of 3 h. Peak C3-20 showed a similar trend as in the glucose pulse perturbation, that is, a rapid decrease after the shift to nitrogen gassing followed by a steady intensity during the 3 h of monitoring. The dynamics of the MCC-IMS signals were very similar to that observed during the imposed Crabtree effect and no new peaks were detected. However, *S. cerevisiae* responded faster to anaerobic than to glucose excess conditions. Changes in the peak intensities of, for example, C3-15 were already observed 7 min after the shift to nitrogen gassing, *i.e.*, before complete anaerobiosis, while the first changes in the MCC-IMS peak intensities after the rapid glucose pulse were observed only after 17 min.

In both perturbation experiments, samples were measured in intervals of 215 s over a period of about 30 min. These rapid measurements show the potential of MCC-IMS analysis for online monitoring and control of bioprocesses. 

### 2.3. Identification of Volatile Metabolites

A disadvantage of MCC-IMS is its poor ability for analyte identification. To get first hints of the mVOC spectrum in the off-gas, we performed HS/SPME GC-MS analyses. Volatile metabolites were extracted from 10 mL the headspace of culture supernatant by solid phase microextraction. Peaks detected in the GC-MS analyses were identified by comparing retention indices and spectra with data from the NIST library (Version 2.0) and the FlavorNet database [32]. Although this identification procedure often results in ambiguous results, it narrowed down the standard measurements conducted with the MCC-IMS for peak identification. 

GC-MS samples were taken from the glucose-limited chemostat cultivation of *S. cerevisiae* CEN.PK during steady state conditions, after perturbation of the continuous cultivation by the glucose pulse and shift to anaerobic conditions (15 min and 50 min after pulse injection and shift to anaerobic conditions, respectively), and of the batch cultivation of the isogenic *ADH1* knock-out mutant during exponential growth and stationary phase. Several peaks in the GC-MS analyses were found in all samples, while some peaks were specific for the different growth conditions or strain used. In total, metabolites could be assigned to 10 peaks by database searches (Table 2). Compounds found in the samples of all culture conditions and if the *S. cerevisiae adh1*∆ mutant were ethanol (in different amounts), 2-pentanone, 2-phenylethanol, 2,3-hexanedione, butyric acid, and isobutyric acid. Benzaldehyde was found in all samples except those from anaerobic cultures. Acetoin and 2,3-butanediol were only detected in the samples of the *S. cerevisiae adh1*∆ strain. Acetoin is an intermediate of the pathway to 2,3-butanediol and can be derived from pyruvate and acetaldehyde via three different pathways [62,63,64,65,66,67]. With the reduced flux of acetaldehyde to ethanol in this mutant, conversion of acetaldehyde to acetoin and further to 2,3-butanediol might be induced as has already been reported for a *ADH1*, *ADH3* and *ADH5* triple knockout mutant of *S. cerevisiae* BY4742 [68]. By contrast, isovaleric acid was detected in all samples expect in those from the *ADH1* knock-out mutant. Besides the 10 metabolites identified in the GC-MS analyses, standards of 1-butanol and acetaldehyde were measured by MCC-IMS. mVOCs were assigned to unknown peaks MCC-IMS signals by comparing the retention time and reduced inverse ion mobility to data of the pure standard substance measurements. As already assumed from the correlation with the ethanol concentration in the fermentation broth, peak C3-15 was identified as ethanol. Of the Ehrlich pathway compounds found in the GC-MS analyses, isobutyric acid, isovaleric acid, and 2-phenylethanol were measured via MCC-IMS. The Ehrlich pathway is a catabolic pathway, which degrades amino acid into aroma compounds such as higher alcohols or volatile fatty acids [35]. The fusel acids (and corresponding alcohols) isobutyric acid, isovaleric acid, and 2-methylbutanoic acid are, for example, derived from the branched chain amino acids valine, leucine, and isoleucine, respectively, by the activity of aldehyde dehydrogenases, encoded by *ALD2* to *ALD6* [38]. Only isobutyric acid could be assigned to one of the peaks (C3-8) detected in the off-gas analyses of yeast fermentations. Unambiguous identification of 2-phenylethanol was not possible due to overlapping peak regions, which is due to co-elution and similar drift times, with 2,3-butanediol. 

The pure standard MCC-IMS measurements identified peak C3-5 as 2-pentanone. In *Penicillium roqueforti*, 2-pentanone is derived from β-oxidation of fatty acids and might be synthesized in *S. cerevisiae* via the same pathway [69]. However, although 2-pentanone has been found in several *S. cerevisiae* fermentations [29,36], a biochemical confirmation of the synthesis via β-oxidation is so far not described. Peak C3-7 (not shown in Figure 2 and Figure 5) was identified as 2,3-hexanedione, which is reported as a metabolite of brewer’s yeast with a cheesy aroma [70] and has recently been detected in the headspace of agar plates cultures of *Corynebacterium glutamicum* [71]. To the best of our knowledge no biosynthetic pathway for this compound has been reported for *S. cerevisiae*.

**Table 2:** Volatile organic compounds detected in fermentations of *S. cerevisiae* growing in glucose minimal salt medium. ND, not detected.

Compound	GC-MS	MCC-IMS	peak	reduced inverse ion mobility 1/K_0_ (Vs cm^−2^)	MCC-IMS retention time (s)	GC-MS retention time (min)
2,3-butanediol	x	x		0.575	4.5	26.517
2,3-hexanedione	x	x	C3-7	0.570	19.4	9.633
2-pentanone	x	x	C3-5	0.554	6.4	5.029
acetoin	x	x		0.532	8.5	16.932
benzaldehyde	x	x		0.566	37.5	25.366
butyric acid	x	x		0.630	24.4	30.127
ethanol	x	x	C3-15	0.516	4.0	4.193
isobutyric acid	x	x	C3-8	0.618	10.4	27.827
isovaleric acid	x	ND		−	−	31.594
2-phenylethanol	x	x		0.578	4.5	39.670

The low recovery rate of metabolites identified by GC-MS might partially be explained by the different sampling procedures. While for the MCC-IMS measurements 10 mL of the off-gas were directly measured, for the GC-MS volatiles were extracted from the culture broth at 50 °C and were preconcentrated. Furthermore, GC-MS peaks were only tentatively identified by comparison with databases and require validation by pure standard analyte measurements. To broaden the spectrum of analyte detection in the MCC-IMS, measurements with both negative and positive ion mode are useful. Our future experiments will focus on the identification of unknown MCC-IMS signals, including thorough verification by GC-MS measurements of pure standard substances. Ideally, feeding experiments with labelled precursors should be performed to conclusively prove whether the identified compounds are actually produced by *S. cerevisiae.*

## 3. Experimental Section

### 3.1. Yeast Strains and Growth Conditions

The yeast strains used in this study were the reference strain *S. cerevisiae* CEN.PK 113-7D [72] and the isogenic *ADH1* knockout mutant *S. cerevisiae* CEN.PK 117 YOL086c::*kan*MX4 [73] devoid of the main alcohol dehydrogenase Adh1p. The yeast strains were grown in Verduyn minimal salt medium [74] containing 20 g L^−1^ glucose, 3 g L^−1^ KH_2_PO_4_, 0.5 g L^−1^ MgSO_4_·7 H_2_O 20.4 g L^−1^ potassium hydrogen phthalate as well as 1 mL of vitamin solution and 1 mL of trace elements. The nitrogen source (NH_4_)_2_SO_4_ was replaced by 2 g L^−1^ urea, as at higher concentrations of ammonia, the water chemistry of the IMS ionisation process could shift to ammonia chemistry [75]. The vitamin solution contained 0.05 g L^−1^ D-biotin, 1 g L^−1^ calcium D-pantothenate, 1 g L^‑1^ nicotinic acid, 25 g L^−1^ *myo*-inositol, 1 g L^−1^ thiamine hydrochloride, 1 g L^−1^ pyridoxine hydrochloride and 0.2 g L^−1^ *p*-aminobenzoic acid. The trace element solution consisted of 15 g L^−1^ EDTA, 4.5 g L^−1^ ZnSO_4_·7H_2_O, 1 g L^−1^ MnCl_2_·4 H_2_O, 0.3 g L^−1^ CoCl_2_·7 H_2_O, 0.3 g L^−1^ CuSO_4_·5 H_2_O, 0.4 g L^−1^ NaMoO_4_·2 H_2_O, 4.5 g L^−1^ CaCl_2_·2 H_2_O, 3 g L^−1^ FeSO_4_·7 H_2_O, 1 g L^−1^ H_3_BO_3_ and 0.1 g L^−1^ KI. All precultures were performed in 500 mL shake flasks filled with 10% medium at 30 °C and 250 rpm. The bioreactor experiments were run in a Sartorius Biostat A plus bioreactor (Göttingen, Germany) with a working volume of 1 L at 30 °C. The fermentation parameters were controlled by an external computer and the software PC Panel μDCU. The pH was monitored with a Mettler Toledo pH electrode and controlled at pH 5 using 10 M potassium hydroxide and 4 M hydrochloric acid. In aerobic fermentations, the fermenter was aerated with pressurized air with a flow rate of 2.8 L min^−1^. To reduce impurities, the air was filtered by a Sartorius Midisart 2000 sterile filter (0.2 µm pore size) and passed through water, resulting in water-saturated gas. Dissolved oxygen concentrations were monitored with a Hamilton Oxyferm dissolved oxygen electrode. If not mentioned otherwise, the dissolved oxygen tension was maintained at 90% by adjusting the stirrer speed. Bioreactor cultivations were started by inoculating to an OD_600_ of 0.1. During batch cultivation, sample for OD and HPLC measurements were taken regularly in the first 12 h. Continuous, glucose-limited cultivations were run at a dilution rate of 0.13 h^−1^ by feeding fresh medium at a flow rate of 2.5 mL min^−1^. A constant volume of culture broth was maintained by positioning a tube at a predetermined height that corresponded to 1120 mL volume and connecting it to a separate pump that removed excess fluid. Both batch and continuous cultivations were single experiments. 

To check for possible contamination during fermentations, samples were examined daily under the microscope (Leica DM750) with a 10X ocular and a 100X oil immersion objective. 

### 3.2. Analytics

The optical density was determined with an Ultrospec 10 photometer (Amersham Bioscience, Amersham, Switzerland) with a fixed wavelength of 600 nm. When necessary, the samples were diluted using demineralized water.

For the determination of glucose and fermentative byproducts, samples were taken directly out of the fermenter using a syringe and a steel pipe. Samples were harvested by centrifugation (Heraeus Megafuge 16R, Thermo Fisher Scientific, Marietta, Ohio, USA) at 5000 rpm for 5 min at 4 °C. The supernatant was stored at −20 °C until further analysis. Analytes were separated using an organic acid resin column (C-S Chromatography, Langerwehe, Germany) at 50 °C. 5 mM H_2_SO_4_ was used as eluent at a flow rate of 0.8 mL min^−1^ (System Gold 125 Solvent Module). Analytes were detected with a UV detector (166 Detector, (Beckman Coulter, Krefeld, Germany) at a wavelength of 210 nm and a RI detector (Melz Differential Refractometer LDC 201) operated at 50 °C. Standard solutions of the analytes were measured in concentrations of 0.1, 0.5, 1, 5, 10, 20, 40 and 50 g L^−1^.

### 3.3. SPME GC-MS Measurements for Validation of Volatile Metabolites

To cross-check the identification of volatile compounds, 10 mL of the supernatant were sampled from the bioreactor and transferred into a headspace vial (20 mL). Metabolites were extracted from the headspace of culture supernatant via solid phase microextraction (CAR/PDMS fibers, Supelco, Steinheim, Germany) and analyzed with GC-MS as described in [41]. Briefly, the samples were incubated at 50 °C for 15 min and after that agitated for 10 s at 250 rpm. Afterwards, the analytes were desorbed in the SPME liner of the GC at 250 °C. GC-MS analyses were carried out with an Agilent 7890A gas chromatograph equipped with an Agilent 7000B triple-quadrupole mass spectrometer (Agilent Technologies, Waldbronn, Germany). The split was set to a ratio of 5:1. The column temperature was increased from 40–250 °C with a rate of 12 °C min^−1^. All other parameters were identical as those described in [41]. For mVOC identification, retention indices and mass spectra were compared with the NIST mass spectral library (Version: 2.0) and data published in the FlavorNet database (flavornet.org, [32]). 

### 3.4. MCC-IMS Measurements

The MCC-IMS used was a BreathDiscovery (B&S Analytics, Dortmund, Germany) with an upstream multi capillary column type OV-5 (Multichrom Ltd. Novosibirsk, Russia) of 17 cm length consisting of approx. 1000 capillaries. The capillaries have an inner diameter of 40 µm and are coated with OV-5 stationary phase with a film thickness of 0.2 µm. The column temperature was set to 40 °C. Samples were ionized with a 550 MBq ^63^Ni ion source. The ionized analytes were introduced into the drift column (length, 120 mm) through a shutter that had a pulse frequency of 50 ms and an opening time of 30 µs. Separation in the drift chamber was carried out in a negative coaxial electric field with an intensity of 300 Vcm^−1^ (positive measurement mode). The MCC-IMS was operated at ambient temperature and pressure (*i.e.*, laboratory conditions). Raw data of mVOCs were acquired using VOCan (B&S Analytik) with a frequency of 10 Hz, 5 consecutive single spectra were averaged. A single round of data acquisition required 0.5 s. The program was used to control all parameters of the MCC-IMS like gas flow and temperature, and to program measurement sequences.

Nitrogen 5.0 (Westfalen, Münster, Germany) was used both as drift gas in the MCC-IMS and as carrier gas in the MCC. The drift gas flow rate was set to 100 mL min^−1^; the carrier gas flow rate was set to 150 mL min^−1^ during batch and 50 mL min^−1^ during continuous fermentation. The fermenter off-gas was introduced into the system through a 10 mL stainless steel sampling loop coupled to a six-port valve. Between single measurements the MCC-IMS and the sampling line was purged with a nitrogen flow of 100 mL min^−1^.

The MCC-IMS topographic plots were evaluated using the software VisualNow (B&S Analytik). Reduced inverse ion mobilities, 1/K_0_ (Vs cm^−2^), were calculated by normalizing the measured drift velocities (drift time per drift distance) to the electric field, temperature and pressure. This reduced ion mobility is characteristic for the ion and independent of the experimental conditions. The program allowed the definition of peak regions and comparison of the peak intensities in different datasets. The intensities of detected peak regions, the reduced ion mobility and retention time were exported to Excel for further data evaluation [76].

The MCC-IMS was connected to the fermenter off-gas with a Teflon tube (ID, 1.58 mm; length, 1000 mm). To prevent overloading of the MCC-IMS, the off-gas was diluted with sterile, moisturized air or nitrogen at a flow rate of 2.4 mL min^−1^. The air was filtered with a 0.2 µm sterile filter. Mixing was achieved by introducing both gas streams into a 500 mL Schott bottle.

### 3.5. Volatile Metabolite Identification

For mVOC identification, pure standard substance measurements were performed, for which the MCC-IMS was operated with identical parameters as during the continuous cultivations. One mL of aqueous standard solutions of a concentration of 0.01 g L^−1^ was filled in 100 mL Erlenmeyer flasks, which were closed with an aluminum cap. The sampling tube was introduced to the flask and the measurement was started.

## 4. Conclusions

Application of MCC-IMS in positive ion mode for online monitoring of fermentation off-gas detected 19 signals produced by *S. cerevisiae* during growth on glucose minimal salt medium. To more comprehensively explore the volatile metabolite spectrum of yeast fermentations, in future experiments IMS measurements in both positive and negative ion mode will be performed. However, although the here presented first analyses probably captured only a minute fraction of *S. cerevisiae*’s volatile metabolite spectrum, they were sufficient to differentiate different *S. cerevisiae* strains and to reveal the impact of different growth conditions on the production of mVOCs. Four compounds were identified by complementary GC-MS measurements of fermentation broth extracts and pure standard substance measurements. Similar to the exometabolome, for which only the main metabolites are usually measured while it is only poorly described systematically [77], only little is known about the synthesis and regulation of volatile metabolites. Even for the well-studied model organism *S. cerevisiae*, the biochemistry of only two of the four mVOCs identified in this study by MCC-IMS and four of the 10 mVOCs identified by GC-MS are well understood. While in this study mVOC synthesis by *S. cerevisiae* was only qualitatively determined, future quantitative analyses will show the significance of mVOC analysis for the description of the cell physiology and its metabolic activity. 
